# Sliding metasurface for wide-angle beam steering with sharp frequency filtering

**DOI:** 10.1038/s41377-026-02422-2

**Published:** 2026-07-27

**Authors:** Haoyang Shi, Xiangming Wu, Xinwei Wang, Yufei Zhao, Jie Tian, Weiren Zhu, Guangwei Hu

**Affiliations:** 1 State Key Laboratory of Radio Frequency Heterogeneous Integration, School of Integrated Circuits, Shanghai Jiao Tong University, Shanghai, China; 2 School of Electrical and Electronic Engineering, Nanyang Technological University, Singapore, Singapore

**Keywords:** Metamaterials, Optical materials and structures

## Abstract

The advancement of electromagnetic technologies necessitates strategies to efficiently exploit spectral and spatial resources in increasingly congested and interference-prone environments. This paper demonstrates, for the first time, a fully passive sliding metasurface that unifies sharp frequency filtering and wide-angle beam steering within a single architecture, thereby establishing a two-dimensional frequency–angle selection window. The proposed metasurface replaces local phase reconfiguration with a global geometric displacement between two complementary phase profiles. This sliding strategy produces a controlled translation of the spectral centroid in reciprocal space, enabling continuous beam steering and suggesting extensibility to other frequency regimes. In the demonstrated implementation, a transmission-line-inspired design is adopted, in which stacked layers are strategically reused to form a quasi–Fabry–Pérot cavity within the meta-atom, enabling simultaneous phase modulation and frequency-selective filtering. Experimental results validate sharp spectral selectivity, ultra-wide out-of-band suppression, and stable beam steering over a ± 57° scanning range. The proposed sliding metasurface offers a compact and broadly applicable platform for next-generation electromagnetic systems, enabling a new modality for coordinated spectral–spatial wave control.

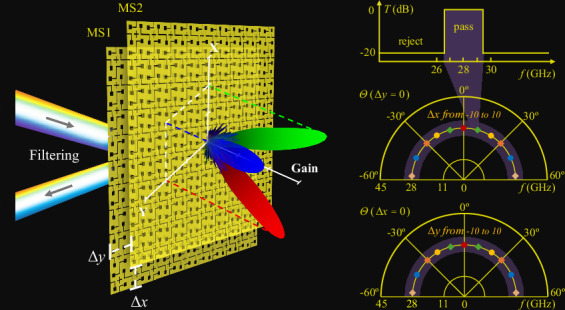

## Introduction

With the rapid proliferation of electromagnetic technologies, efficient utilization of spectral and spatial resources has become increasingly critical, calling for advanced wave-manipulation strategies^1–5^. Metasurfaces, with their ability to tailor electromagnetic waves at subwavelength scales, have emerged as a promising approach for engineering wave–matter interactions, thus providing a distinctive solution^6–8^. These surfaces have been widely applied in microwave and optical domains, where their multidimensional control of transfer functions—such as phase, polarization, and frequency—enables the full exploitation of wave degrees of freedom, thereby advancing applications including information transmission, sensing, and computation^9–16^. For instance, metasurfaces can realize frequency filtering and beam steering, thereby enhancing selectivity and multiplexing across spectral and angular domains, which in turn maximizes channel utilization, mitigates interference, and supports large-scale integrated systems^17–22^.

Conventional metasurface-based beamforming employs phase-gradient arrays to precisely control beam direction in transmission or reflection modes^23–25^. To achieve dynamic beam steering, active elements such as PIN diodes are typically integrated to reconfigure the phase distribution, thereby enhancing spatial division multiplexing^26–28^. However, these designs often lack a sharp transition between the passband and stopband. With increasing spectral congestion and functional integration, traditional designs fail to discriminate multi-frequency waves, leading to poor spectral selectivity and cross-band interference. A promising solution is to embed frequency windowing into metasurface–wave interactions by engineering appropriate resonance conditions and interlayer coupling^29–31^. Resonant layers can modulate displacement current flux to emulate transmission-line behavior and thereby construct field-based circuit filters^32–34^. For example, cascading metasurfaces in accordance with higher-order Butterworth filters enables sharp bandpass filtering and strong out-of-band suppression^35^. Nevertheless, existing spatial filtering schemes focus on frequency, neglecting other wave dimensions and thereby limiting the realization of multidomain control.

In this paper, we propose a dual-layer sliding metasurface that establishes a two-dimensional frequency–angle selection window by integrating frequency filtering with wide-angle beam steering. Beam steering is enabled through the lateral displacement between two complementary effective phase profiles, which produces a translation of the spectral centroid in reciprocal space and consequently a controlled deflection of the output beam. By replacing local phase reconfiguration with a global geometric displacement, the proposed strategy eliminates the need for meta-atom-level dynamic phase tuning designed for specific frequencies. As a result, the sliding mechanism is inherently geometry-driven and can, in principle, be extended to other frequency regimes. In the demonstrated metasurface operating at 26.5–29.5 GHz (corresponding to the 5G n257 band), frequency selectivity is achieved through meta-atom configurations inspired by transmission-line filter designs. The stacked meta-layers are strategically reused to embed quasi-Fabry–Pérot cavities, enabling discrete 4-bit phase modulation while maintaining high-performance frequency filtering. Moreover, the metasurface is entirely passive, requiring no active components or external driving circuits, thereby ensuring fabrication simplicity and high damage resistance^36,37^. Experimental results demonstrate effective passband selectivity, ultra-wide out-of-band suppression (20 dB), and wide-angle beam steering over a ± 57° scanning range. The proposed sliding metasurface therefore provides a compact, energy-efficient, and scalable platform for joint frequency–angle domain control, offering a new modality for passive wave manipulation across diverse wave-based systems.

## Results

### Design of the Metasurface

**Fig. 1:**
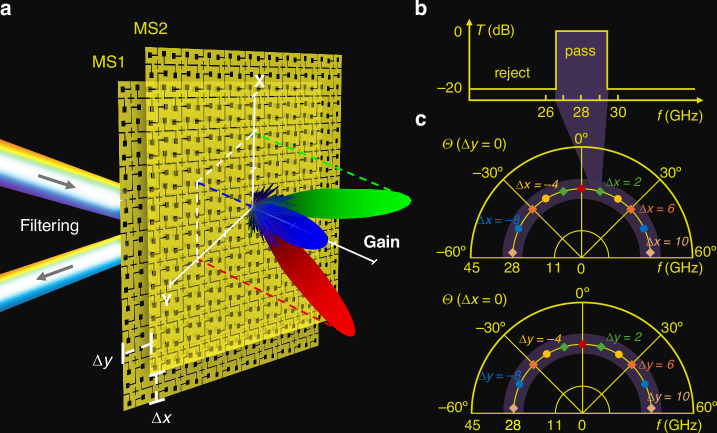
Conceptual Representation of the Sliding Metasurface for Integrated 2D Beam Steering and Frequency Filtering. **a** Beam deflection along the x-axis, y-axis, and oblique directions controlled by lateral displacements. **b** The frequency-selective characteristics of the sliding metasurface. **c** Beam steering angle within the passband as a function of relative lateral displacement, quantified by the number of shifted meta-atoms

Figure 1 presents the schematic of the sliding metasurface platform, which establishes a two-dimensional frequency–angle selective electromagnetic interface with a dynamically tunable angular range. Lateral displacement of the lower metasurface (MS2) relative to the upper layer (MS1) introduces a controlled phase gradient across the aperture, thereby deflecting the transmitted beam. In the demonstrated implementation, MS1 adopts the proposed filtering–phase integrated metasurface design, while MS2 is required only to function as a phase-only metasurface with a matching aperture. Figure 1a illustrates that the metasurface allows arbitrary in-plane translation within the XOY plane, enabling beam steering along the x-axis, y-axis, and off-axis directions. As shown in Fig. 1c, deflection angles increase monotonically with the meta-atom displacement count. This passive mechanism achieves continuous beam control without active circuit components. Concurrently, the metasurface functions as a bandpass filter, transmitting signals within the 26.5–29.5 GHz while suppressing out-of-band interference across a broad frequency range (Fig. 1b).

**Fig. 2:**
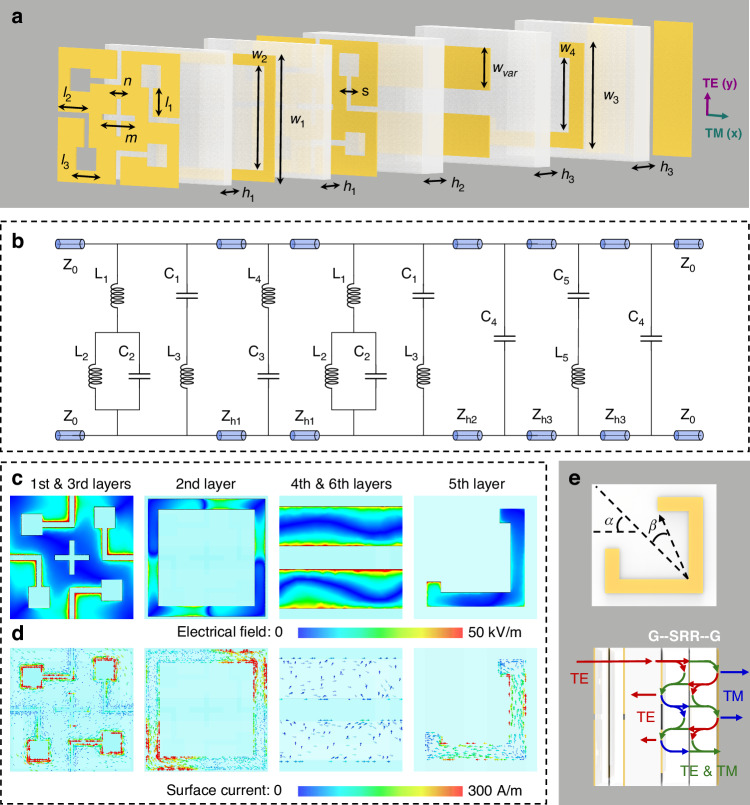
Meta-Atom Structure and Its Transmission-Line Representation with Phase-Control Mechanism. **a** Schematic diagram of the meta-atom structure. **b** Equivalent transmission-line filter circuit of the multilayer stacked meta-atom. **c** Electric field and **d** surface current distribution of the multi-layer structure, illustrating the realization of structured inductive and capacitive components. **e** Schematic and phase control principle diagram of the Fabry–Perot-like cavity

To co-control spectral and angular properties, each meta-atom can be interpreted as a transmission-line filtering structure with embedded phase modulation. To elucidate the underlying working mechanism, an equivalent model is introduced as a physical interpretation, in which metasurface resonances modulate near-field displacement currents and establish field-based circuit analogues. Vertically stacked resonant layers form a spatially distributed high-order filter, where multilayer coupling sharpens frequency selectivity and enhances out-of-band rejection. As shown in Fig. 2a, each meta-atom integrates six patterned metallic layers and five dielectric spacers, forming a compact stack with a 3.5 mm periodicity and a total thickness of 2.63 mm. The equivalent transmission-line circuit representation is illustrated in Fig. 2b. Figure 2c, d show the simulated energy distributions, highlighting how specific physical patterns give rise to inductive and capacitive behaviors in the equivalent circuit. The coiled structures in Layers 1 and 3 enhance the effective inductance and capacitance: narrow gaps concentrate electric fields, producing capacitive effects, while the elongated current paths along the metallic traces give rise to inductive behavior. These effects are represented by the first and third branch sections of the transmission-line circuit. The closed ring in Layer 2 forms a standard *L*_4_*C*_3_ resonator. Layers 4, 5, and 6 comprise orthogonal metallic gratings and an interleaved open ring. The gratings concentrate electric fields (*C*_4_), while the open ring supports resonant current loops (*L*_5_*C*_5_), collectively increasing the filter order and further refining frequency selectivity. Specifically, the dielectric substrates have thicknesses of ℎ_1_ = 0.254 mm and *h*_3_ = 0.508 mm, with an effective relative permittivity of 2.65. The central dielectric substrate is polymethacrylimide (PMI) foam with a thickness of ℎ_2_ = 1 mm. Table 1 summarizes the detailed physical geometries, whereas the lumped component values corresponding to Fig. 2b are given in Supplementary Material 1. Overall, this vertically stacked strategy establishes a clear physical correspondence between the metasurface and its circuit interpretation, providing physical insight and design guidance for realizing high-order, multifunctional metasurface responses.

**Table 1:** Detailed geometric dimensions of the patterned layers

Parameter	Value (mm)	Parameter	Value (mm)
*l*_1_	0.9	*w*_1_	3.3
*l*_2_	0.9	*w*_2_	2.72
*l*_3_	0.6	*w*_3_	2.75
*m*	0.97	*w*_4_	1.95
*n*	0.15	*s*	0.15

Building upon the multilayer filtering model, the lower grating–split-ring–grating arrangement is further exploited as a nested quasi-Fabry–Perot cavity for transmission phase modulation. As illustrated in Fig. 2a, the cavity comprises front (x-oriented) and rear (y-oriented) metallic gratings. For a TE-polarized incident wave, the front grating enables efficient coupling into the cavity, whereas the rear grating suppresses co-polarized transmission, forcing the field to undergo multiple internal reflections. During each round trip, the split-ring resonator (SRR) embedded between the two gratings partially converts the co-polarized field into an orthogonal (TM-polarized) component. Only this cross-polarized component can be transmitted through the rear grating, thereby forming a polarization-selective output channel. Consequently, the transmitted field results from the coherent superposition of cross-polarized waves generated over successive internal oscillations, reflecting repeated cavity interactions rather than a single-pass process.

The phase imparted to the converted wave is governed by two independent geometric degrees of freedom of the SRR, namely the split orientation α and the opening angle β, which introduce controllable phase offsets during each polarization-conversion event. This geometric phase control enables the SRR to provide stable phase biases within the transmission passband, as detailed in Supplementary Material 3. Moreover, all coding states share an identical cavity configuration with a similar spacer thickness and grating arrangement, such that the frequency-dependent phase contribution associated with cavity propagation is common to all states. As a result, the relative phase differences of the converted waves remain stable across the passband. Through the combined action of polarization-selective cavity interference and geometry-controlled phase bias, the proposed metasurface realizes a 4-bit phase-encoding scheme with quasi-continuous phase modulation, while preserving the filtering performance with negligible additional insertion loss and minimal footprint expansion.

### Numerical validation and analysis

**Fig. 3:**
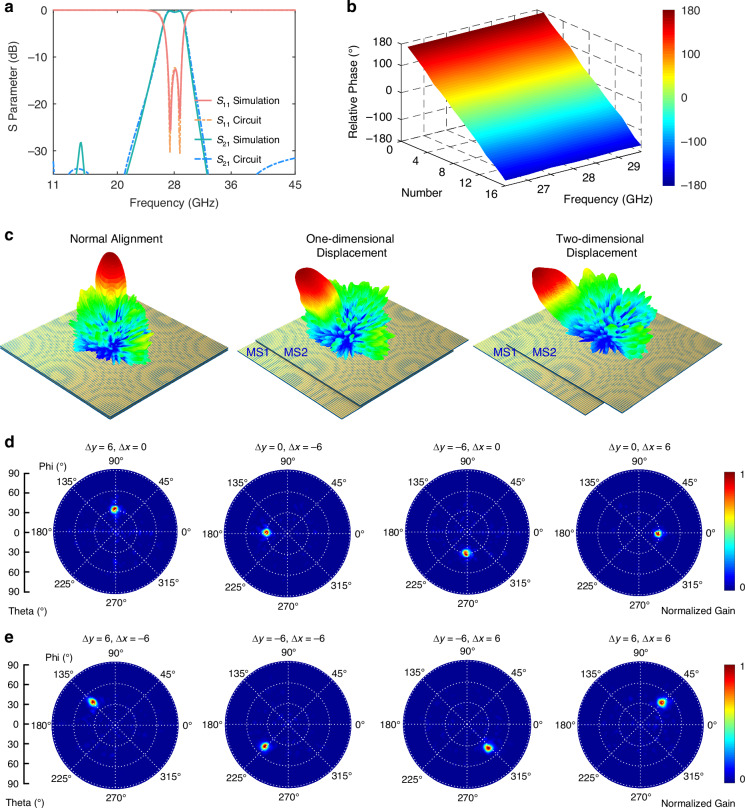
Performance Analysis and Far-Field Demonstration of 1D/2D Beam Steering via Sliding Metasurface. **a** Comparison of the scattering curves predicted by the equivalent circuit model and the numerical simulation results. **b** Phase gradient map showing the variation with frequency and meta-atom number. **c** Schematic diagrams of the dual-layer metasurface structure and the corresponding beam deflection principle under different relative positions: fully aligned, laterally shifted along one axis (1D sliding), and diagonally shifted along both axes (2D sliding). **d** Far-field radiation pattern for beam scanning at a 30° deflection angle. **e** Far-field radiation pattern for oblique beam scanning at a 45° deflection angle

To validate the proposed design strategy, full-wave simulations were performed in CST Microwave Studio 2023. The unit cell was modeled with periodic boundary conditions in the x–y plane, and Floquet ports were applied along the z axis. Figure 3a compares the simulated transmission coefficient with the equivalent circuit model, showing excellent agreement and confirming that the electromagnetic response of the meta-atom is well captured by the transmission-line theory. A transmission loss of less than 1 dB is achieved from 26.5 to 29.5 GHz (n257 band), corresponding to a relative bandwidth of 10.7%. The return loss remains lower than –10 dB within the passband, indicating efficient impedance matching. Moreover, ultra-wide out-of-band suppression surpassing 20 dB is observed on both sides of the passband, covering the 11–45 GHz range. The 4-bit phase coding scheme is implemented by varying the opening angle and orientation of the split-ring resonators. Figure 3b illustrates the relative phase within the passband as a function of frequency and coding index. A monotonic phase gradient is clearly observed, and importantly, the phase differences between coding states remain relatively stable across the passband. This stable phase progression originates from the nested quasi-Fabry–Perot cavity, which accumulates controlled phase delays through internal reflections and cross-polarization conversion. The geometric variations and corresponding amplitude–phase responses of the coding elements are summarized in Table 2, illustrating that phase discretization is determined by the split-ring orientation angle *α* and the opening angle *β*, while grating width *w*_*var*_ tunes amplitude uniformity. For all coding states, the transmission loss remains below 1 dB.

**Table 2:** Structural Parameters, Amplitude, and Phase of 16 Meta-Atoms for 4-bit Phase Encoding

^Num^	^α°^	^β°^	^w^_var_	^A (dB)^	^φ°^	^Num^	^α°^	^β°^	^w^_var_	^A(dB)^	^φ°^
^1^	^45^	^42.4^	^1.1^	^-0.98^	^180^	^9^	^135^	^42.4^	^1.1^	^-0.86^	^0^
^2^	^45^	^36.2^	^1.1^	^-0.57^	^157.5^	^10^	^135^	^36.2^	^1.1^	^-0.41^	^-22.5^
^3^	^45^	^32.3^	^1.2^	^-0.59^	^135^	^11^	^135^	^32.3^	^1.2^	^-0.47^	^-45^
^4^	^45^	^29.0^	^1.3^	^-0.43^	^112.5^	^12^	^135^	^29.0^	^1.3^	^-0.39^	^-67.5^
^5^	^45^	^26.6^	^1.4^	^-0.24^	^90^	^13^	^135^	^26.6^	^1.4^	^-0.23^	^-90^
^6^	^45^	^20.7^	^1.4^	^-0.31^	^67.5^	^14^	^135^	^20.7^	^1.4^	^-0.29^	^-112.5^
^7^	^45^	^14.9^	^1.4^	^-0.47^	^45^	^15^	^135^	^14.9^	^1.4^	^-0.46^	^-135^
^8^	^45^	^9.3^	^1.4^	^-0.42^	^22.5^	^16^	^135^	^9.3^	^1.4^	^-0.42^	^-157.5^

To achieve continuous beamforming on a passive compact platform, we implement beam deflection via controlled lateral misalignment between dual metasurface layers. In Fourier optics, the interaction of an incident electromagnetic field with a metasurface corresponds to a convolution of the input spectrum *E*_in_ (*k*_*x*_, *k*_*y*_) with the Fourier transform of the metasurface’s spatially varying transmission function:

1 $${\tilde{E}}_{{\rm{out}}}({k}_{x},{k}_{y})=\tilde{T}({k}_{x},{k}_{y})\ast {\tilde{E}}_{{\rm{in}}}({k}_{x},{k}_{y})$$

where $\tilde{T}({k}_{x},{k}_{y})$ denotes the reciprocal-space modulation imposed by the metasurface, whose envelope is determined by the intrinsic structural design of the metasurface. The proposed sliding mechanism does not reshape this envelope, but primarily translates it in reciprocal space, thereby shifting the angular pass window. Specifically, this translation is achieved by displacing the spectral centroid of $\tilde{T}({k}_{x},{k}_{y})$ from (0, 0) to a non-zero wavevector$(\delta {k}_{\varDelta x},\delta {k}_{\varDelta y})$, resulting in a corresponding beam deflection, as described by Eq. (2).

2 $${\theta }_{x}={\rm{arcsin}} \left(\frac{\delta {k}_{{\Delta }x}}{{k}_{0}}\right)\,{\rm{or}}\,{\theta }_{y}={\rm{arcsin}} \left(\frac{\delta {k}_{{\Delta }y}}{{k}_{0}}\right)$$

For simultaneous displacements along both axes, the beam direction is determined by the vector composition of the two orthogonal wave-vector shifts. We realize this *k*-space displacement using a dual-layer phase-modulation metasurface, with each layer imparting a complementary parabolic phase profile:

3 $${\varphi }_{1}(x,y)=-{\varphi }_{2}(x,y)=A({x}^{2}+{y}^{2})$$

Perfect alignment cancels the phase contribution, resulting in a spectrum concentrated at the *k*-space origin. However, introducing lateral displacement (Δ*x*, Δ*y*) between the layers, the resultant transmission function acquires a linear phase component:

4 $$\begin{array}{c}{\varphi }_{total}(x,y)=A({x}^{2}+{y}^{2})-A({(x+\varDelta x)}^{2}+{(y+\varDelta y)}^{2})\\ =-2A(\varDelta x\cdot x+\varDelta y\cdot y)-A(\varDelta {x}^{2}+\varDelta {y}^{2})\end{array}$$

where $\varDelta x$ and $\varDelta y$ represent the translation distance. Although the displacement is inherently continuous, it is represented in the following analysis as discrete offsets quantified by the number of shifted unit cells for a more intuitive presentation. The modulation induced by lateral sliding becomes equivalent to multiplying the real-space transmission function of the entire metasurface by a phase factor:

5 $$t(x,y)={t}_{0}(x,y){e}^{-i2A(\varDelta x\cdot x+\varDelta y\cdot y)-i{\phi }_{0}}$$

where ${t}_{0}(x,y)$ denotes the transmission function in the aligned state, ${e}^{-i2A(\varDelta x\cdot x+\varDelta y\cdot y)}$ represents the induced phase gradient, ${\phi }_{0}=A(\varDelta {x}^{2}+\varDelta {y}^{2})$ is a global constant phase term introduced by the sliding displacement. Taking the Fourier transform, the corresponding transmission function in *k*-space becomes:

6 $$\tilde{T}({k}_{x},{k}_{y})={e}^{-i{\phi }_{0}}{\tilde{T}}_{0}({k}_{x}-2A\cdot \varDelta x,{k}_{y}-2A\cdot \varDelta y)$$

Under the Fourier-transform convention adopted here, the spectral-centroid shift is given by

7 $$\delta {k}_{\varDelta x}=2A\cdot \varDelta x,\delta {k}_{\varDelta y}=2A\cdot \varDelta y$$

Therefore, the sliding operation produces a rigid translation of the angular spectrum, resulting in controlled beam deflection as described by Eq. (2). Reversing the transform convention only reverses the sign definition of the wave-vector shift, without affecting the steering magnitude. In this work, plane-wave illumination is adopted to analyze the proposed sliding-based beam-steering mechanism, with the phase coefficient *A* set to 7.0 × 10^3 ^m^−2^. Detailed phase maps for the two metasurface layers are provided in Supplementary Material 4. Non-ideal illuminations with slowly varying amplitude and weak phase curvature introduce only minor smoothing through angular-spectrum convolution and do not alter the underlying mechanism. This sliding strategy realizes beam steering through relative geometric translation between metasurface layers, fundamentally differing from programmable metasurfaces that rely on dynamic modification of local meta-atom phase states. The resulting global, geometry-driven phase modulation mechanism avoids band-specific phase-switching designs, thereby improving scalability across different frequency bands. In principle, this sliding-based approach can be extended to different frequency regimes, provided that similar dual-layer configurations can be designed to realize the required phase profiles at the target frequency.

Figure 3c illustrates a schematic of beam-steering control via lateral sliding of the dual-layer metasurface. With the layers fully aligned, the incident wave propagates without angular deviation. Lateral misalignment along the x and y axes introduces a linear phase shift, which displaces the wavevector centroid in reciprocal space and thereby deflects the transmitted beam. Figure 3d shows the two-dimensional polar beam patterns of the metasurface for displacements along the x and y directions, where $\varDelta x$ and $\varDelta y$ represent unit-cell displacements along the respective axes. Sliding along the x and y directions steers the beam within the XOZ and YOZ planes. At moderate deflection angles, such as 30 degrees, the beam exhibits strong main-lobe energy concentration with negligible sidelobes. For oblique sliding directions, beam deflection results from the vector summation of wavevector shifts along the *x* and *y* axes. Figure 3e shows the far-field radiation pattern under 45° diagonal beam deflection, achieved with a lateral displacement of 6 units along both *x* and *y* axes. While theoretical deflection angles derive from vector composition of horizontal and vertical offsets, simulation observations reveal minor main-lobe deviations—the central angle diverges ≤ 2° from the theoretical 45°. At 45° deflection, the far-field energy distribution exhibits marginally greater divergence compared to the 30° case, yet maintains strong directivity with focused beam characteristics. Beam steering along the off-axis diagonal direction, corresponding to equal displacements along the x and y axes, can reach up to approximately 70°, as detailed in Supplementary Material 6. This extensive scanning range facilitates advanced detection and imaging applications^38,39^. However, steering at large angles beyond 57 degrees reduces angular resolution, as the main lobe broadens over elevated sectors ranging from 60 to 80 degrees. This behavior is mainly attributed to the reduction of the effective radiating aperture, which arises both from the limited physical aperture of the present implementation during large lateral sliding and from general finite-aperture constraints common to all planar beam-steering systems. For a sufficiently large metasurface aperture, the sliding-based beam-steering mechanism does not introduce additional degradation in large-angle scanning performance beyond these general finite-aperture effects.

### Experimental demonstrations

**Fig. 4:**
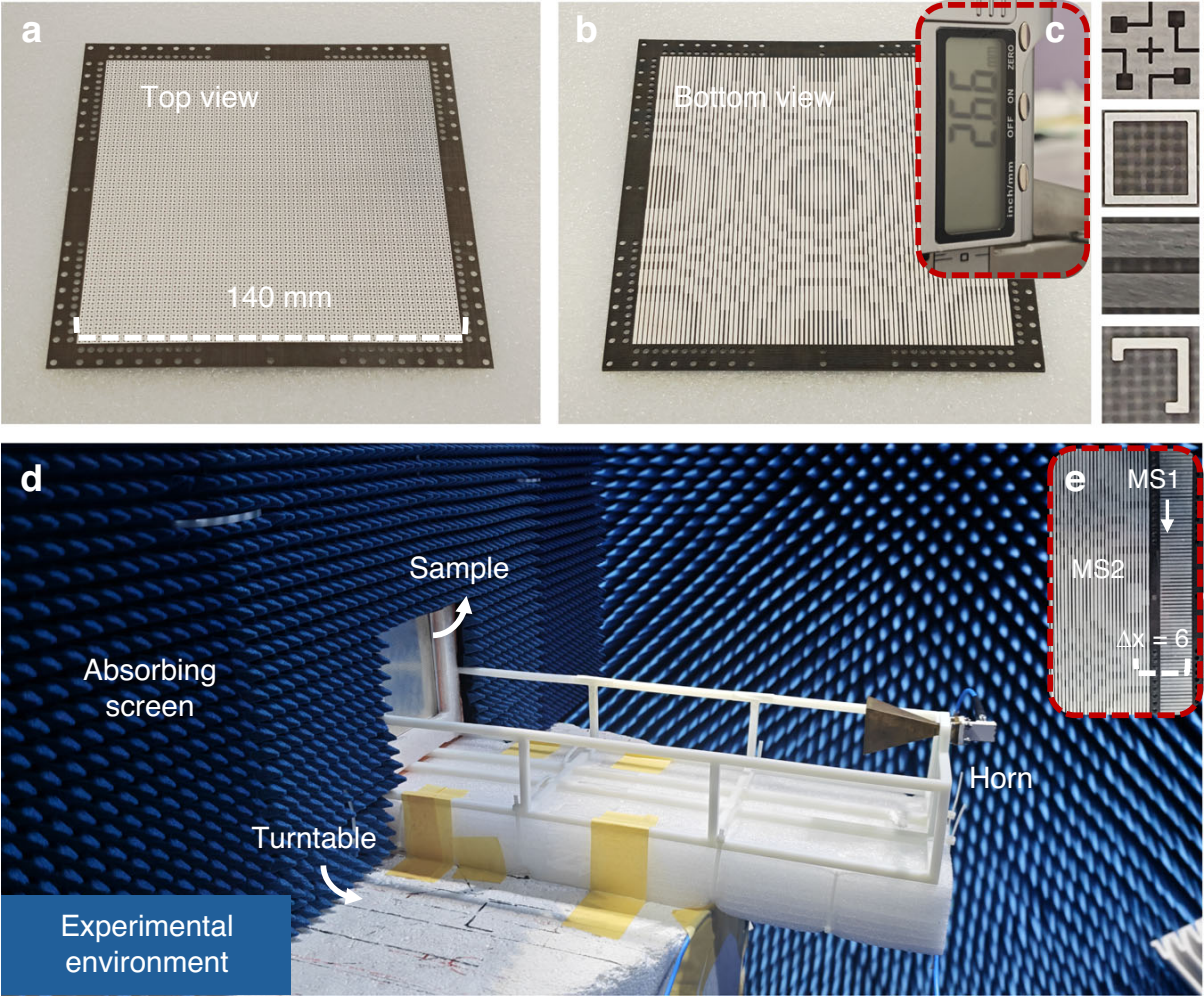
Fabricated Sample and Experimental Setup. **a** Top view and **b** bottom view of the filtering-phase metasurface sample. **c** Thickness of the metasurface. **d** Experimental environment and measurement setup. **e** Schematic illustration of a 6-unit relative shift between MS1 and MS2 along the x-direction

To validate the effectiveness of the proposed strategy for joint frequency and angular control of electromagnetic waves, a metasurface sample consisting of a 40 × 40 unit array is fabricated via standard printed circuit board (PCB) technology. The fabricated sample closely matches the simulated model in structure and geometry, with top and bottom views shown in Fig. 4a, b. It measures 140 mm × 140 mm in-plane with a thickness of 2.66 mm. A slight thickness deviation of 0.03 mm from the design value is observed, which can be attributed to typical material thickness tolerances and fabrication variations. Detailed views of subwavelength meta-atom highlight metallic patterns across layers. Scattering parameters and far-field radiation patterns are characterized within an indoor microwave chamber, as shown in Fig. 4d. The system integrates two metasurface layers and a feeding horn antenna mounted horizontally on a turntable using a 3D-printed support frame for angular sweep. The separation between the two metasurface layers is set to 1 mm. An absorptive screen was placed around the sample to suppress edge effects, unwanted diffraction, and direct leakage bypassing the metasurface. Figure 4e presents a 6-unit relative displacement along the x-direction during experimental validation. In the proposed sliding architecture, MS2 is only required to provide transmissive phase modulation covering the operating band, as frequency-selective functionality does not require repeated implementation in such systems.

**Fig. 5:**
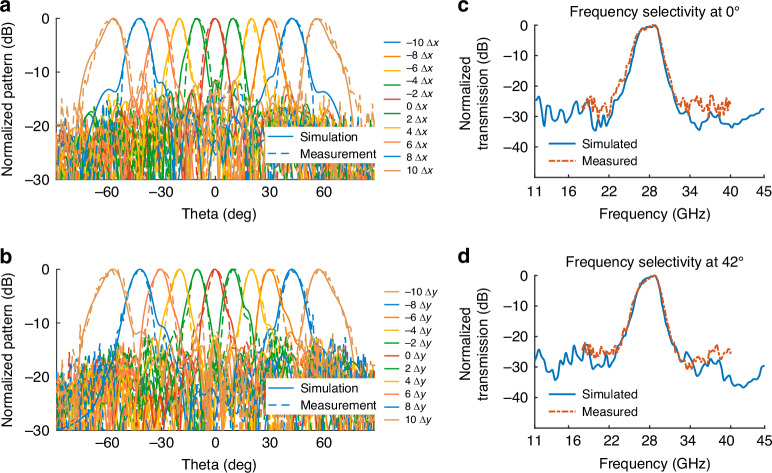
Two-dimensional Beam Steering and Frequency-selective Characteristics. The lateral displacement between the sliding metasurfaces enables two-dimensional beam steering in both the **a** X and **b** Y axes. Frequency selectivity of the metasurface for two representative beam directions: **c** 0° and **d** 42°

Figure 5a, b compares the simulated and measured radiation patterns under various lateral displacements, demonstrating the wide-angle beam-scanning capability enabled by the proposed sliding strategy. As described by the beamforming principle, off-axis deflection arises from the vector superposition of wavevector shifts along orthogonal directions. Accordingly, the experimental characterization focuses on beam steering along the x- and y-directions to validate the metasurface’s capability for continuous two-dimensional scanning. As the relative displacement increases from 0 to 10 units, the main-lobe direction scans from 0° to 57°, taking discrete values of 0°, 10°, 20°, 30°, 42°, and 57°. Both simulations and measurements confirm that well-defined beams are formed at all steering angles, with sidelobe levels remaining below −10 dB. The measured half-power beamwidth gradually broadens from 9° to 16° as the scanning angle increases. In practical measurements, minor angular deviations may arise from unavoidable assembly imperfections, such as slight sample tilting and residual misalignment in the illumination.

Figure 5c, d illustrates the frequency-dependent transmission at two representative steering angles, which reflects the overall frequency-selective behavior of the sliding metasurface. Both simulation and measurement demonstrate the formation of an effective passband within the n257 band, along with a consistent trend of out-of-band suppression. When evaluated at a fixed observation angle, the frequency responses exhibit moderate fluctuations within the passband and variations in the scattering background in the stopband. These features originate from finite-aperture truncation and edge effects, which redistribute part of the electromagnetic energy into undesired angular components. In practical measurements, such effects lead to reduced out-of-band suppression compared to simulations. Nevertheless, the metasurface still maintains effective suppression over two frequency intervals, spanning 18–24.1 GHz and 31.3–40 GHz, with suppression levels on the order of 20 dB. Experimental validation is limited to 18–40 GHz by the measurement setup, while additional full-wave simulations indicate that the stopband extends over an ultra-wide frequency range from 11 GHz to 45 GHz. Overall, both simulations and experimental results demonstrate that the proposed sliding metasurface enables effective spectral and spatial manipulation of electromagnetic waves, featuring sharp frequency selectivity and dynamically tunable beamforming. This capability facilitates two-dimensional control and channel allocation of electromagnetic wave propagation, providing a versatile platform for managing wave transmission in both frequency and angular domains.

## Discussion

The sliding metasurface presented here provides a passive route to coordinating two fundamental degrees of freedom of electromagnetic waves: frequency and propagation direction. In contrast to conventional metasurfaces for beam steering, in which dynamic wavefront control commonly relies on meta-atom-level phase reconfiguration, the proposed strategy converts local phase tuning into a global geometric translation between two complementary phase profiles. This mechanism reduces the reliance on active components and biasing circuits, while maintaining continuous beam steering over a wide angular range. More importantly, since the steering principle is governed by the relative spatial phase relationship rather than by a frequency-specific electrical tuning mechanism, it may be transferred to other electromagnetic regimes, provided that suitable phase surfaces can be fabricated.

As a multidimensional strategy for passive wave control, the proposed metasurface enables a two-dimensional frequency–angle selection window, with the capability to discriminate and modulate waves in both spectral and angular domains. In the microwave demonstration, the integration of frequency selectivity and phase modulation is enabled by a transmission-line-inspired meta-atom design, where the stacked layers are simultaneously reused to construct a high-order filtering response for sharp frequency selectivity and an embedded quasi–Fabry–Pérot cavity for phase control. This paradigm for constructing multidimensional transfer functions is particularly relevant to interference suppression, spectral-spatial multiplexing, and electromagnetic front ends, where unwanted signals may differ from desired signals in either spectral or angular coordinates. The proposed sliding metasurface therefore introduces a passive modality for multidomain wave manipulation in diverse wave-based systems.

## Methods

### Numerical simulation

This study employs the commercial software CST Microwave Studio 2023 for physical modeling and full-wave simulations. During the design and performance evaluation of the meta-atoms, the frequency-domain solver is used in conjunction with unit cell boundary conditions. Dual-polarized Floquet ports are applied to excite the meta-atom, enabling the extraction of the scattering parameter matrix under both horizontal and vertical polarizations. E-field and H-field monitors were used to visualize the electric-field distributions and surface-current patterns of the resonant elements, thereby clarifying their correspondence with the lumped components in the equivalent transmission-line filter model. The metasurface array is constructed through a co-simulation and optimization process combining MATLAB and CST. The phase distribution, determined by the proposed analytical formulation, is computed in MATLAB and then mapped onto the CST model to guide the physical layout of the metasurface. For the full-structure simulation of the metasurface, open boundary conditions are applied, and appropriate local mesh refinements are manually configured to ensure accuracy. Minor variations in fine spectral ripples may occur due to mesh refinement and convergence criteria, but they do not affect the reported conclusions.

### Experimental Setup

The frequency-selective and beam-scanning performance of the proposed sliding metasurface prototype was experimentally characterized in a microwave chamber. The metallic layers of the metasurface were fabricated using standard PCB copper with a thickness of 18 μm. The overall measurement configuration is shown in Fig. 4d. Two linearly polarized horn antennas were employed as the transmitting and receiving antennas, respectively. The transmitting horn was mounted on a 3D-printed holder and connected to Port 1 of a vector network analyzer (VNA), while the receiving antenna was connected to Port 2. To suppress edge effects and direct leakage bypassing, the metasurface sample was embedded within a windowed absorptive screen, realized by mounting microwave absorbers onto a foam board. In addition, metallic copper foil was applied to the foam and supporting frame surrounding the sample to further suppress unintended transmission outside the effective aperture. The transmitting horn was positioned sufficiently far from the sample to provide an approximately uniform incident field over the effective aperture used in the measurement. Prior to measurement, the background response without the metasurface sample was recorded as a reference. The final results were obtained by normalizing the measured data with respect to this reference.

## Supplementary information

Sliding metasurface for wide-angle beam steering with sharp frequency filtering

## Data Availability

All data that support the findings of the study are available from the corresponding authors upon reasonable request.
