# Supplementary material for: Sliding metasurface for wide-angle beam steering with sharp frequency filtering
Source: Light Sci Appl. 2026 Jul 27;15:332. doi: 10.1038/s41377-026-02422-2 (PMC13402743; doi:10.1038/s41377-026-02422-2)
Supplement: Supplementary file 1 — Sliding metasurface for wide-angle beam steering with sharp frequency filtering [file 41377_2026_2422_MOESM1_ESM.pdf]

Supplementary Information for

# Sliding metasurface for wide-angle beam steering with sharp frequency filtering

Haoyang Shi[1,2], Xiangming Wu[1], Xinwei Wang[2], Yufei Zhao[2], Jie Tian[1], Weiren Zhu[1*] and Guangwei Hu[2*]

*[1*] State Key Laboratory of Radio Frequency Heterogeneous Integration, School of Integrated Circuits, Shanghai Jiao Tong University, Shanghai, 200240, China.*

*[2*] School of Electrical and Electronic Engineering, Nanyang Technological University, 50 Nanyang Avenue, 639798, Singapore*

[*]Correspondence to: guangwei.hu@ntu.edu.sg, weiren.zhu@sjtu.edu.cn

**Keywords:** wide-angle beam steering, frequency-selective filtering, passive metasurface, reconfigurable metasurface, multidimensional wave control

## 1. Detailed high-order transmission-line filter parameters

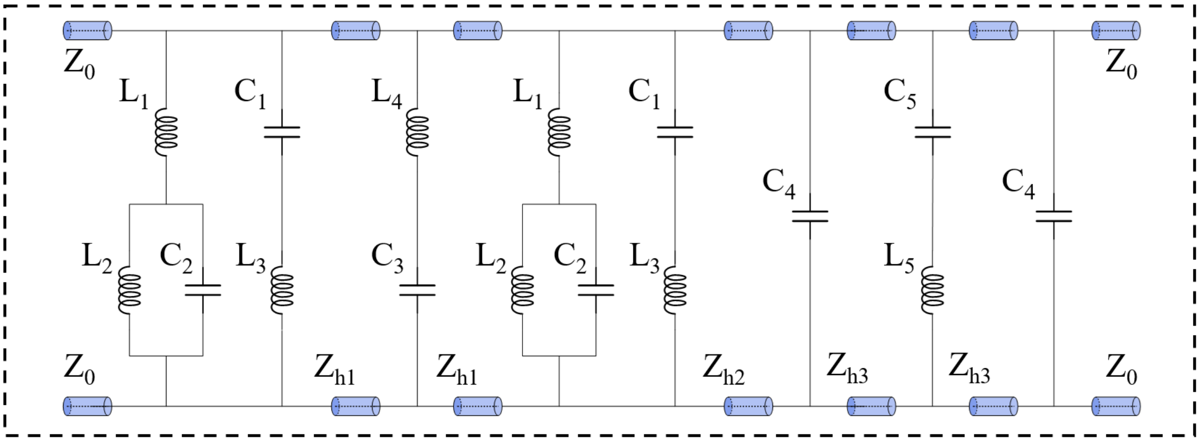


**Figure S1.** Detailed high-order transmission-line filter structure and circuit parameters.

By designing the resonance conditions, the metasurface can modulate the displacement current flux in the near field, effectively emulating the behavior of a transmission line in controlling electromagnetic waves or fields. Building upon this principle, we established the transmission-line model for the filter emulated by the metasurface, as shown in Figure S1. The input and output port impedances are set to the air wave impedance of 377Ω. During the circuit design process, we simultaneously considered both the resonant structure of the meta-atom and the potential phase-control mechanisms. The filter is composed of a multi-stage series and parallel LC circuits that are coupled with each other. The transmission lines represent the dielectric layers between the resonant structures of the meta-atom. All component parameters of the circuit are listed in Table S1.

Table S1: Circuit parameters of the transmission-line filter

| Capacitance | Value | Inductance | Value |
|---|---|---|---|
| $C_1$ | 25 fF | $L_1$ | 4.7 nH |
| $C_2$ | 0.27 pF | $L_2$ | 2.5 nH |
| $C_3$ | 0.28 pF | $L_3$ | 0.85 nH |
| $C_4$ | 0.02 pF | $L_4$ | 0.24 nH |
| $C_5$ | 0.2 pF | $L_5$ | 0.93 nH |

## 2. Detailed meta-atom design and geometric parameters

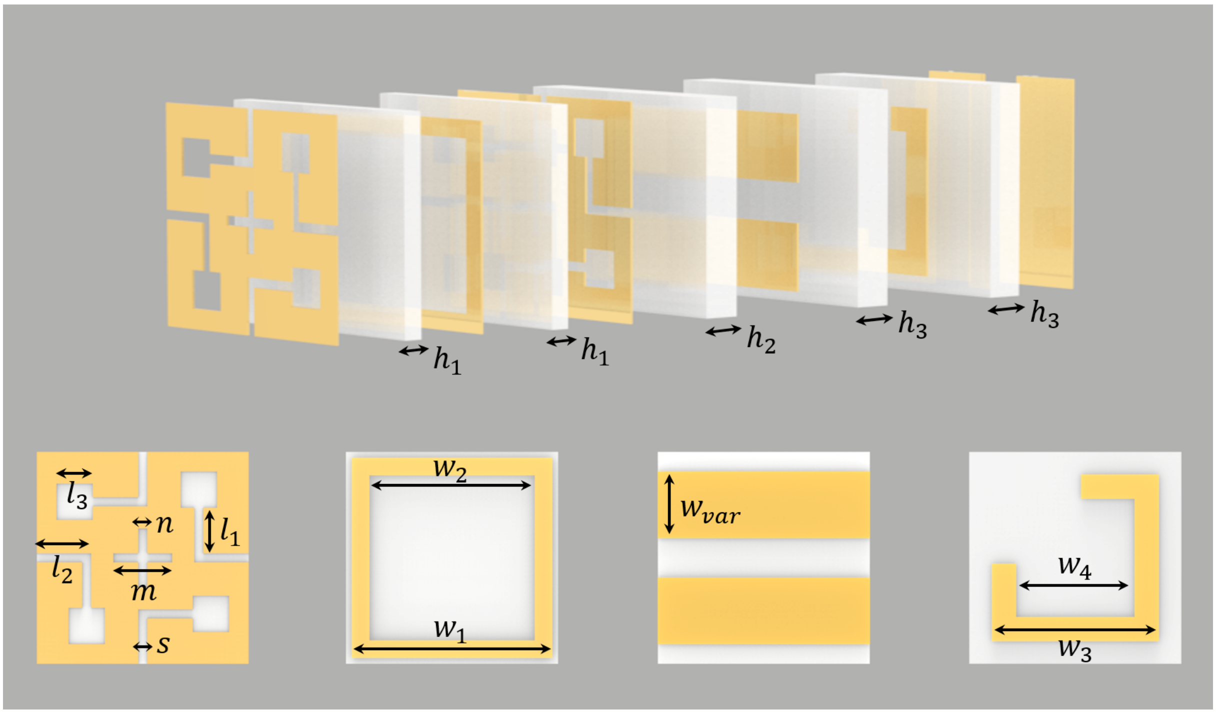


**Figure S2.** Schematic of the metasurface structure and the geometric dimensions of each patterned layer.

The meta-atom consists of a multilayer coupled structure that simultaneously enables sharp frequency filtering and precise control over the transmission phase of electromagnetic waves. The first and third metallic layers feature intricately coiled patterns designed to enhance both capacitive and inductive effects. The second layer adopts a classical ring-shaped resonator structure. Layers four to six are configured in a grating–split-ring–grating (G–SRR–G) arrangement. Together, these six patterned layers are engineered with carefully tuned resonant modes and interlayer coupling, forming a field-driven high-order spatial filter. The dielectric substrates have an effective relative permittivity of 2.65 and thicknesses of $h_1$=0.254 mm and $h_3$=0.508 mm, respectively. The central dielectric substrate is a PMI foam layer with low permittivity and a thickness of $h_2$=1 mm. The periodical length of the metasurface is $p$=3.5 mm. The variable grating width $w_{var}$ and the aperture size of the split-ring resonators are provided in Table 1 of the main text, while the geometric dimensions of the remaining structural patterns are listed in Supplementary Table S2.

Table S2: Detailed geometric dimensions of the patterned layers

| Parameter | Value (mm) | Parameter | Value (mm) |
|---|---|---|---|
| $l_1$ | 0.9 | $w_1$ | 3.3 |
| $l_2$ | 0.9 | $w_2$ | 2.72 |
| $l_3$ | 0.6 | $w_3$ | 2.75 |
| $m$ | 0.97 | $w_4$ | 1.95 |
| $n$ | 0.15 | $s$ | 0.15 |

## 3. Phase Degrees of Freedom and Phase Stability of the Phase Tuning Mechanism.

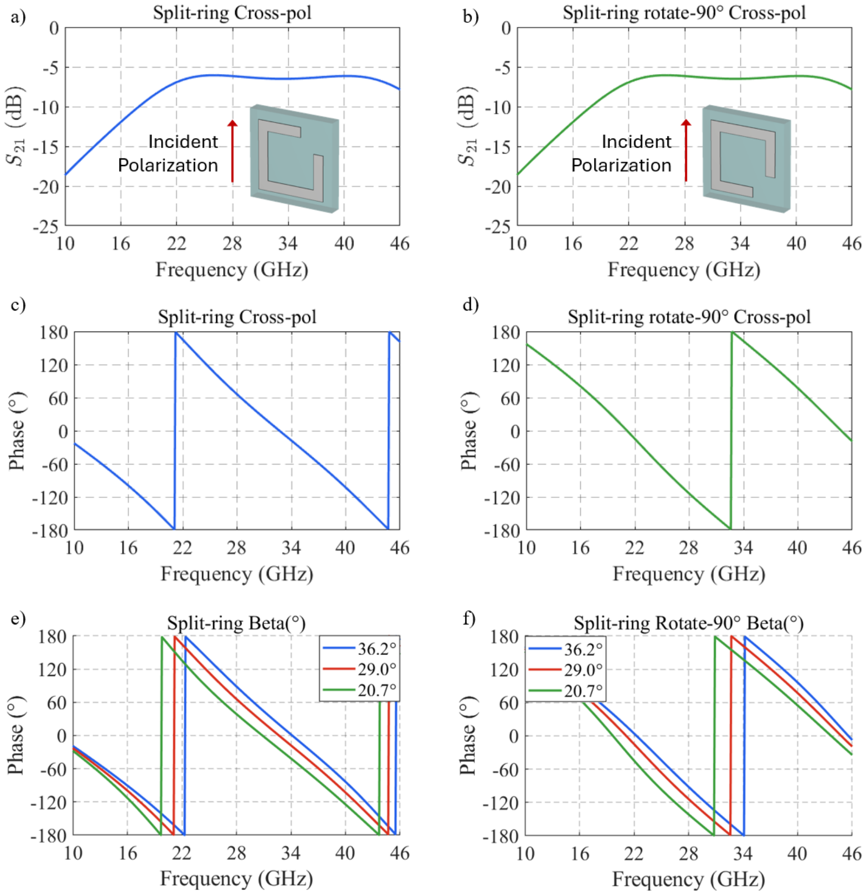


**Figure S3.** Cross-polarized transmission ($S_{21}$) of the split-ring resonator for orientations of (a) 45° and (b) 135° with respect to the principal polarization. For identical SRR geometries, orientations of 45° and 135° with respect to the incident polarization, corresponding to panels (c) and (d), introduce a stable 180° phase difference in the converted cross-polarized wave. Panels (e) and (f) show that varying the opening angle $\beta$ provides continuous phase tuning for the two SRR orientations.

In the proposed metasurface, the degrees of freedom for phase modulation primarily originate from the geometric parameters of the split-ring resonator (SRR), namely the split orientation and the opening angle of the ring. To elucidate the physical origin of these phase-tuning freedoms, we first examine the transmission response of an isolated SRR layer in a qualitative manner, which serves to reveal the intrinsic phase control capability provided by the SRR geometry.

A well-known electromagnetic characteristic of SRRs is that, when the split orientation is not aligned with the incident polarization direction but instead forms a finite angle, part of the incident co-polarized wave can be converted into a cross-polarized component. As summarized in Table 1 of the manuscript, for coding states Num 1–8, the split orientation of the SRR forms

an angle of 45° with respect to the incident polarization, while for Num 9–16, the corresponding angle is 135°. Figures S3(a) and (b) present the transmission coefficients of the cross-polarized channel for SRRs with these two split orientations, demonstrating that both configurations enable effective polarization conversion from the incident wave into the cross-polarized channel.

We further analyze the transmission phase associated with the cross-polarized component for these two split orientations. In Figs. S3(c) and S3(d), it is observed that rotating the SRR by 90° results in a robust phase difference of approximately 180° in the transmitted cross-polarized wave. This behavior constitutes the first degree of freedom for phase modulation introduced by the SRR orientation. In addition, as shown in Figs. S3(e) and S3(f), varying the opening angle of the SRR also induces a controllable phase shift in the cross-polarized transmission. This phase variation associated with the opening angle represents the second degree of freedom for phase modulation.

When the SRR is embedded within the multilayer filtering structure to form the grating–split ring–grating (G–SRR–G) configuration, its intrinsic phase-tuning degrees of freedom are incorporated into the cavity-assisted transmission process. In this configuration, the polarization-selective gratings establish a cavity in which only the cross-polarized components generated by the SRR can be transmitted. As a result, the output phase is determined by the cumulative phase offsets imposed by the SRR during successive polarization-conversion events, rather than by a single-pass interaction. The detailed round-trip process is illustrated schematically in Fig. 2(e) in the main text.

Regarding the stability of the transmission phase within the passband, Figs. S3(c)–S3(f) show that the phase offsets imparted by the SRR through variations in split orientation and opening angle remain nearly linear and stable over the target frequency range of 26.5–29.5 GHz. Moreover, for all coding states Num 1–16, the cavity spacing defined by the G–SRR–G structure remains identical, ensuring that all coding states share a similar cavity condition. As a result, while the absolute transmission phase varies with frequency, the relative phase differences determined by the SRR geometry remain stable across the entire passband, enabling the realization of 16 distinct and robust phase-coding states.

## 4. Detailed phase maps of the MS1 and MS2

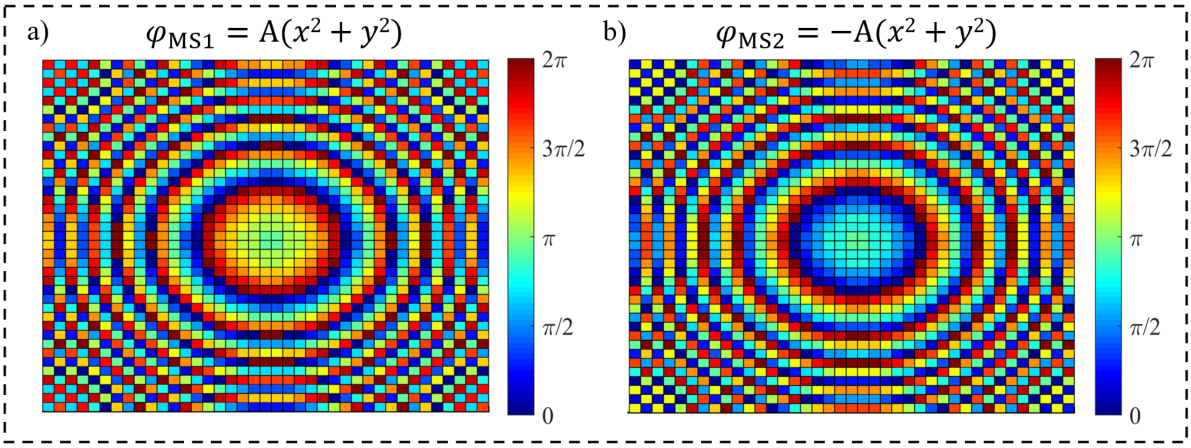


**Figure S4. Detailed Phase Profiles of MS1 and MS2.** (a) Parabolic (quadratic) phase distribution of MS1. (b) Parabolic (quadratic) phase distribution of MS2, designed to be complementary to MS1.

To enable the introduction of a linear phase shift through lateral displacement between the layers, the phase profiles of MS1 and MS2 were designed as fully complementary parabolic (quadratic) functions, as shown in Figures S4(a) and S4(b).

$$\varphi_1(x,y) = -\varphi_2(x,y) = A(x^2 + y^2) \qquad \text{(S1)}$$

Here, $A$ denotes the tuning constant of the phase distribution and is set to $7.0 \times 10^3$ $m^{-2}$ in this work. According to Eqs. (2) and (7) in the main text, different values of $A$ determine the shift of the spectral centroid of the metasurface modulation function in $k$-space under the same displacement distance, and consequently, the resulting beam deflection angle.

## 5. Phase variation introduced by lateral displacement

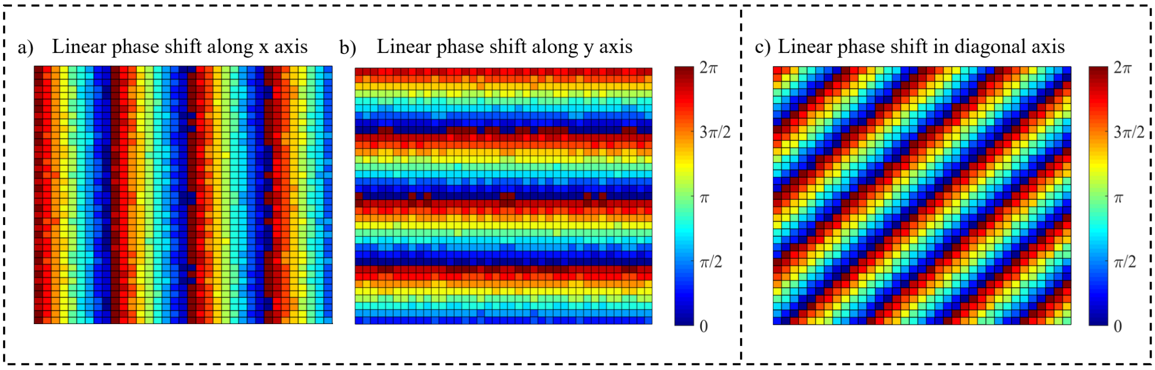


**Figure S5-1. Phase Variation Introduced by Lateral Displacement.** Phase variations induced by relative sliding along the (a) x-axis and (b) y-axis. (c) Phase variation under diagonal sliding.

Figure S5-1 illustrates the linear phase variations introduced by the relative displacement between the two metasurface layers. These variations arise from the superposition of two parabolic (quadratic) phase profiles after lateral shifting, as described in Equation (S2):

$$\begin{aligned}\varphi_{total}(x,y) &= A(x^2+y^2) - A((x+\Delta x)^2+(y+\Delta y)^2) \\ &= -2A(\Delta x \cdot x + \Delta y \cdot y) - A(\Delta x^2 + \Delta y^2)\end{aligned} \tag{S2}$$

The phase variation induced by diagonal displacement, shown in Figure S5-1(c), is essentially the vector sum of the phase gradients along the x and y directions. This enables continuous beam steering along arbitrary in-plane directions.

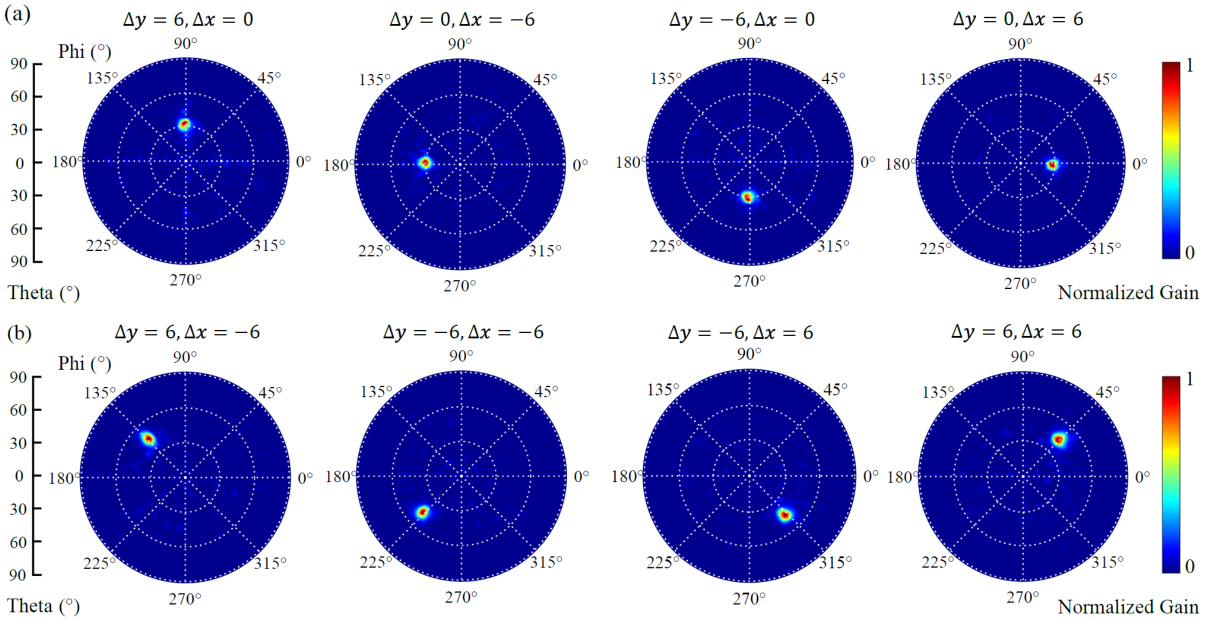


**Figure S5-2. Diagonal Wavevector Shift as a Vector Sum of x- and y-Axis Components.** (a) Far-field radiation patterns of transmitted beams deflected along the x and y axes. (b) Far-field radiation pattern of the

transmitted beam deflected along the diagonal direction, corresponding to the vector sum of x- and y-axis deflections.

The diagonal phase variation can be constructed by superimposing the phase gradients along the x and y axes. Likewise, beam deflection in any arbitrary direction follows the same principle: the shift of the transmitted wavevector center can be synthesized by vector addition of wavevector shifts along the x and y directions. For instance, the 45° deflection observed in Figure S5-2(b) can be achieved by combining two 30° deflections along the horizontal and vertical axes. Experimentally, this vector composition is directly reflected in the additive lateral displacement distances along the x and y axes.

## 6. Radiation patterns under extreme-large deflection angles.

Figure S6 illustrates the far-field radiation pattern of the sliding metasurface at extreme beam deflection angles. As shown in the figure, the main lobe of the deflected beam is centered around 70°, with a relatively broad half-power beamwidth spanning a large range of elevation angles $\theta$. In particular, at azimuth angles φ = 225° and 315°, the main lobe extends over a wide $\theta$ range from 60° to 80°. This significant broadening of the main lobe and the degradation of directionality arise from the limited effective aperture and strong angular dispersion at large deflection angles. Under such conditions, the spatial selectivity and angular resolution of the system are substantially reduced, and the metasurface primarily ensures energy coverage rather than precise directional transmission.

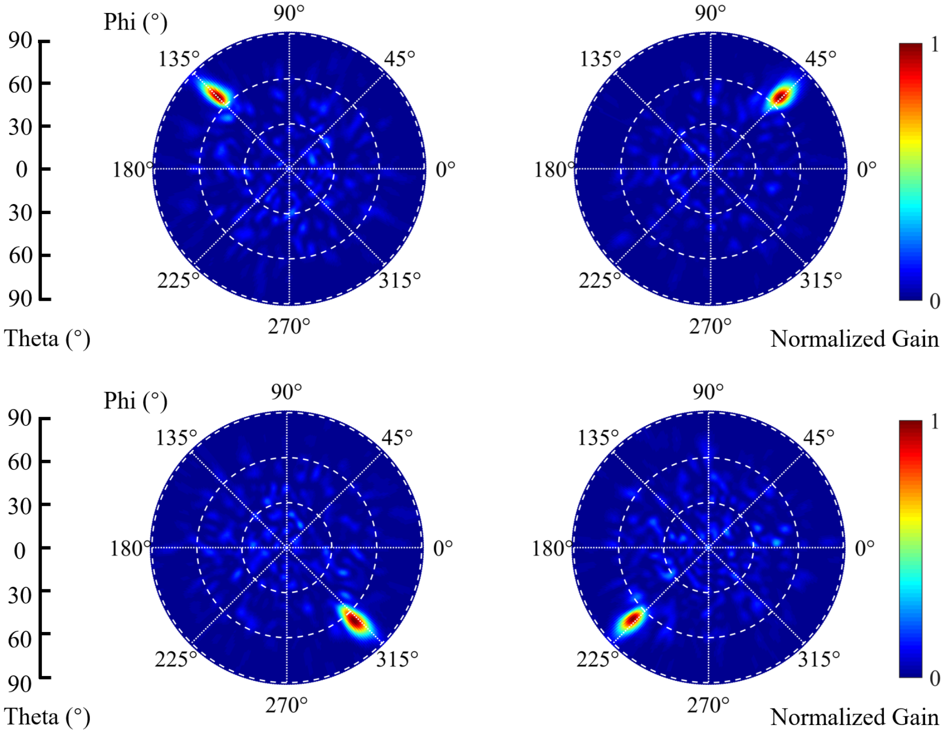


**Figure S6.** Farfield radiation patterns under extreme-large deflection angles.

## 7. Tolerance and Practical Analysis

### 7.1 Influence of Interlayer Separation

The interlayer separation between the two metasurface layers plays a critical role in both the frequency-selective response and the sliding-based beam steering performance.

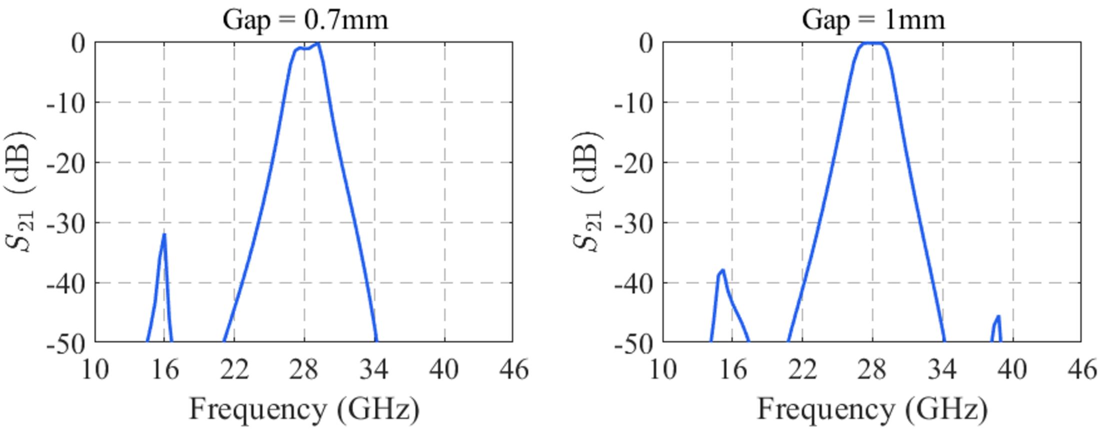


**Figure S7-1.** When the distance between the two metasurface layers becomes too small, near-field coupling perturbs the intended frequency-selective filtering behavior, resulting in a deviation of the transmission amplitude from the designed response.

When the separation becomes too small, near-field coupling between the two metasurfaces can no longer be neglected. In the present implementation, the transmission amplitude remains stable at a separation of 1 mm in the simulated cases, whereas noticeable deviations emerge when the distance is reduced to approximately 0.7 mm, as shown in Fig. S7-1. At such gaps, near-field coupling perturbs the intended frequency-selective filtering behavior, resulting in distortion of the transmission amplitude.

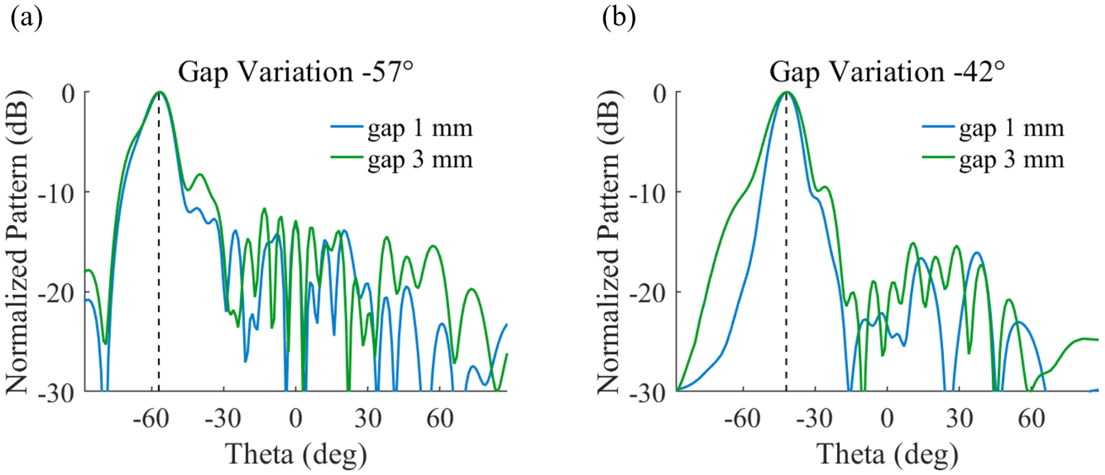


**Figure S7-2.** Influence of the interlayer separation on beam pointing accuracy and beam quality at large and

sensitive steering angles.

Conversely, excessive interlayer separation degrades the beam-forming quality of the sliding-based steering mechanism. As shown in Fig. S7-2, increasing the interlayer gap from 1 mm to 3 mm progressively broadens the main lobe and worsens sidelobe levels. At the maximum steering angle of 57° (Fig. S7-2(a)), a 3 mm separation still preserves accurate beam pointing but causes evident main-lobe broadening and sidelobe elevation. At a moderate steering angle of 42°, the degradation becomes much less pronounced, indicating that beam quality is more sensitive to interlayer spacing at extreme deflection angles. To maintain acceptable performance across the full scanning range (−57° to 57°), the interlayer separation should be kept below 3 mm.

This degradation arises because beam steering is enabled by the relative sliding of two designed phase profiles that jointly synthesize a continuous aperture phase gradient. When the separation becomes large, free-space propagation introduces increased diffraction between the metasurface layers, disturbing their geometric phase alignment. The dominant *k*-space spectral-centroid translation mechanism described by Eqs. (1)– (7) in the main text remains valid, and beam pointing accuracy is preserved. However, the enlarged spacing introduces additional propagation-induced diffraction components not included in the idealized model, which reduce aperture phase coherence and degrade beam quality, resulting in main-lobe broadening and elevated sidelobes.

Therefore, to avoid frequency-selective degradation caused by interlayer coupling and beam-scanning deterioration induced by interlayer diffraction, the separation is practically constrained to 1–3 mm in the present implementation as a balanced design choice.

### 7.2 Robustness under oblique incidence and interlayer relative tilt

The stability of the sliding metasurface under oblique incidence and interlayer angular misalignment is further examined.

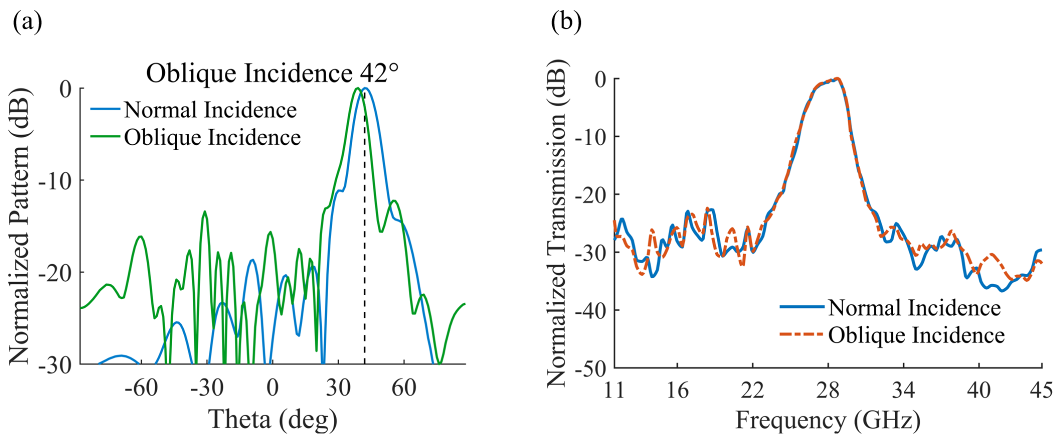


**Figure S7-3.** (a) Beam-steering deviation at large deflection angles and (b) array frequency-selective response variation of the sliding metasurface under 3° oblique plane-wave incidence.

Figure S7-3 shows the simulated beam patterns and frequency-selective responses under a 3° oblique plane-wave incidence. A slight tilt of the incident wave introduces a corresponding offset in the steered beam at a large deflection angle (42°), together with minor beam broadening and moderate sidelobe elevation. This effect is caused by the additional linear phase gradient imposed across the aperture under oblique illumination, which alters the effective phase distribution through superposition with the designed phase profile and consequently shifts the beam direction. The spectral response under oblique incidence remains stable, with only a slight variation in the stopband trend (Fig. S7-3(b)).

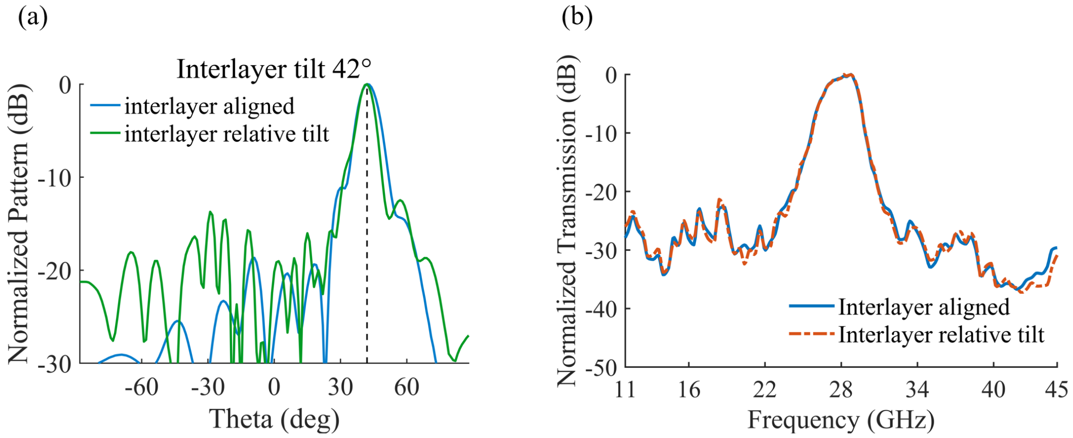


**Figure S7-4.** (a) Beam-steering deviation at large deflection angles and (b) array frequency-selective response variation of the sliding metasurface under interlayer misalignment (relative tilt between the two metasurfaces).

Figure S7-4 illustrates the effect of interlayer angular misalignment, modeled as a relative tilt between the two metasurfaces. The interlayer tilt angle is approximately 1°. Beam pointing

accuracy is largely preserved, while moderate beam distortion and sidelobe increase are observed (Fig. S7-4(a)). The frequency-selective characteristics remain nearly unchanged under this condition (Fig. S7-4(b)).

Overall, the two-dimensional frequency–angle selection functionality is maintained under both oblique incidence and interlayer tilt. The beam offset under oblique illumination follows conventional phased-aperture behavior and is physically predictable.

### 7.3 Practical Implementation

In practical implementations, fabrication tolerances and experimental imperfections may introduce deviations from the ideal design parameters. For the proposed sliding metasurface, interlayer misalignment mainly manifests as a small perturbation to the intended relative displacement between the two metasurface layers. Since beam steering is governed by lateral translation of the overall phase profile across the aperture, such misalignment primarily leads to a slight offset in the beam pointing direction, without altering the beam shape or the underlying steering mechanism, as described in Equations (S3) and (S4). *dx* and *dy* represent the unintended interlayer misalignment.

$$\begin{aligned}\varphi_{total}(x,y) &= A(x^2+y^2) - A((x+\Delta x+dx)^2+(y+\Delta y+dy)^2) \\ &= -2A\left[(\Delta x+dx)\cdot x+(\Delta y+dy)\cdot y\right] - A\left[(\Delta x+dx)^2+(\Delta y+dy)^2\right]\end{aligned} \tag{S3}$$

$$\delta k_{\Delta x} = 2A\cdot(\Delta x+dx)\ ,\quad \delta k_{\Delta y} = 2A\cdot(\Delta y+dy) \tag{S4}$$

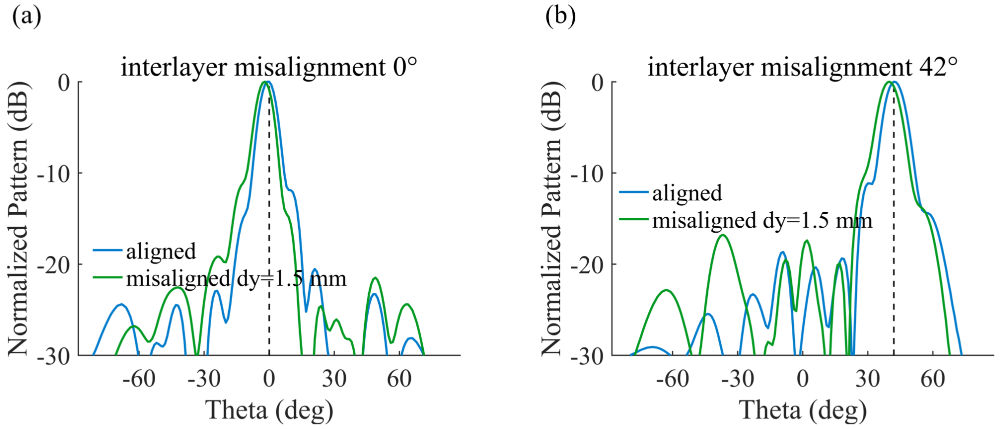


**Figure S7-5.** Effect of interlayer misalignment on the beam pointing accuracy.

The influence of interlayer lateral misalignment on beam-steering accuracy was also

examined through full-wave simulations. Representative steering conditions, including normal radiation (0°) and a large steering angle (42°), were considered. As shown in Fig. S7-5(a), a 1.5 mm unintended misalignment along the y-direction introduces a pointing deviation of about 2° under normal radiation, while the beam profile remains largely unchanged. For the large-angle steering case corresponding to an intended displacement of $8\Delta y$ (producing a 42° deflection in the YOZ-plane), the same displacement error results in a ~2° reduction of the steering angle due to insufficient sliding displacement, as shown in Fig. S7-5(b). In practical implementations, the interlayer misalignment is typically well below 1 mm, and the resulting pointing deviation remains within an acceptable tolerance. Therefore, interlayer misalignment introduces only a minor and predictable perturbation to the beam-steering performance.

Moderate fluctuations in the relative permittivity result in a small shift of the passband center frequency, while the transmission characteristics remain otherwise stable. Importantly, slight dielectric-parameter variations affect all coding elements in a similar manner and therefore preserve the relative phase relationships among different coding states. As the sliding-based beam steering relies on the relative translation of the global phase distribution, such variations do not compromise the beam steering functionality.

In the sliding metasurface architecture, MS1 implements the filtering–phase integrated meta-atom design proposed in this work, which constitutes the key technical contribution. By contrast, MS2 serves only to provide the phase profile required for the sliding operation, as dictated by the theoretical model in Eqs. (1)– (7). Specifically, MS2 is required to support broadband transmissive phase modulation fully covering the passband. In principle, it can be realized using any efficient phase-modulating metasurface with high transmission over the operating frequency range. As a representative example, the Fabry–Pérot-like G–SRR–G configuration discussed in the main text and Supplementary Material is referenced as one possible implementation of MS2. It should be emphasized that, in the context of the filtering–phase integrated meta-atom design, the G–SRR–G structure is an integral component that actively participates in the coupled resonant optimization of the overall frequency response, as described in Fig. 2 in the main text and in the analysis of the frequency-selective mechanism. In that role, it forms part of a multi-order filtering architecture designed to enhance spectral

selectivity. When the G–SRR–G structure is extracted and employed independently as a phase-only metasurface for MS2, its design does not require consideration of the multi-layer coupling and spectral engineering associated with the integrated filtering–phase configuration. Consequently, the parameter design becomes significantly more flexible. As discussed previously, this structure is capable of supporting the required 4-bit phase encoding and therefore serves as a convenient reference implementation for MS2, rather than a mandatory design choice.

For practical systems such as communication base stations or radar platforms, the lateral displacement between metasurface layers can be realized using well-established mechanical translation solutions, including motorized linear stages or guided translation mechanisms commonly used in microwave and antenna engineering. The achievable response speed is primarily determined by the actuation system rather than by the metasurface itself and can be tailored to specific application requirements. For many practical scenarios, such as sector-based beam steering or adaptive beam reconfiguration, mechanical tuning is typically sufficient. Moreover, the proposed architecture is inherently robust due to its fully passive and circuit-free nature. Since the structure consists only of metallic and dielectric components, long-term stability and repeatable operation can be ensured using standard mechanical engineering measures, such as precision guides and locking mechanisms.
